# Correction: Associations between continuity of primary and specialty physician care and use of hospital-based care among community-dwelling older adults with complex care needs

**DOI:** 10.1371/journal.pone.0258708

**Published:** 2021-10-12

**Authors:** Aaron Jones, Susan E. Bronskill, Hsien Seow, Mats Junek, David Feeny, Andrew P. Costa

The hazard ratio label on the first line of Fig 2 is incorrect. The ratio should read “0.90 (0.89–0.92)”. The authors have included a corrected version below.

**Fig 2:**
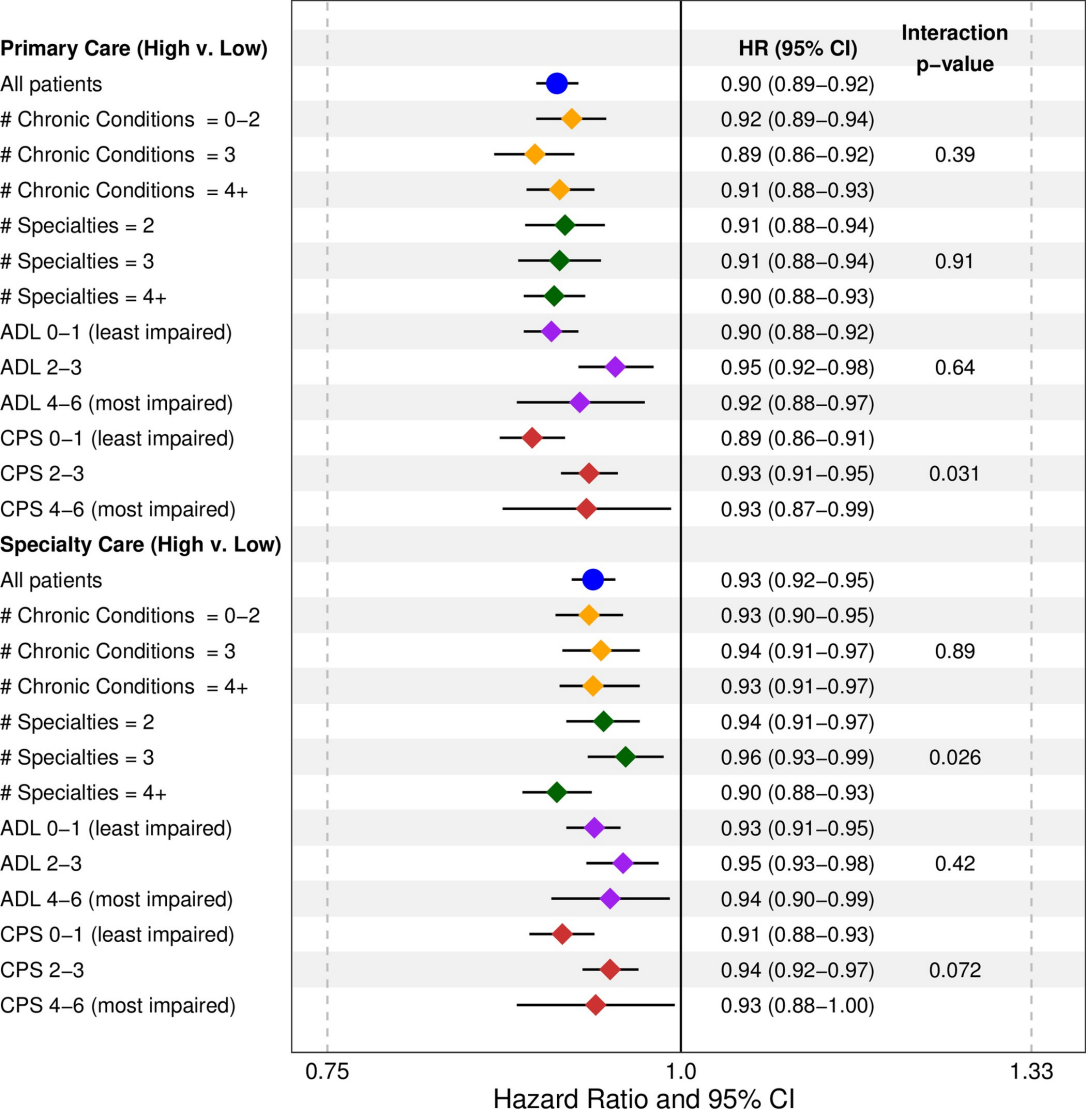
Associations between continuity of care and risk of an emergency department visit across effect modifiers.
