# Supplementary material for: Associations between continuity of primary and specialty physician care and use of hospital-based care among community-dwelling older adults with complex care needs
Source: PLoS One. 2020 Jun 19;15(6):e0234205. doi: 10.1371/journal.pone.0234205 (PMC7304563; doi:10.1371/journal.pone.0234205)
Supplement: S1 Appendix — (PDF) [file pone.0234205.s001.pdf]

Appendix 1: Databases used in the study

**ICES Databases**	**Description**
Ontario Health Insurance Policy Claims (OHIP)	The OHIP claims database contains information on inpatient and outpatient services provided to Ontario residents eligible for the province's publicly funded health insurance system by fee-for-service health care practitioners (primarily physicians) and "shadow billings" for those paid through non-fee-for-service payment plans. The main data elements include patient and physician identifiers (encrypted), code for service provided, date of service, associated diagnosis, and fee paid.
National Ambulatory Care Reporting System (NACRS)	The NACRS is compiled by the Canadian Institute for Health Information and contains administrative, clinical (diagnoses and procedures), demographic, and administrative information for all patient visits made to hospital- and community-based ambulatory care centres (emergency departments, day surgery units, hemodialysis units, and cancer care clinics). At ICES, NACRS records are linked with other data sources (DAD, OMHRS) to identify transitions to other care settings, such as inpatient acute care or psychiatric care.
Home care Database(HCD)	The HCD is a clinical client centric database that captures all services that are provided by or coordinated by Community Care Access Centres (CCACs). The data elements captured include information on: client, intake, assessment, admission & discharge, diagnosis and surgical procedure, and care delivery. ICES receives home care data from the Ontario Ministry of Health and Long-Term Care (MOHLTC). The primary purpose of the information collected through the HCD is to aid in planning and better clinical insight into clients who encounter service through CCACs.
Resident Assessment Instrument (RAI)-Home care source (OACCAC)	The RAIHC database is managed by the Community Care Access Centres (CCACs) and is a standardized clinical assessment to all long-stay home care clients in Ontario defined as clients receiving ongoing support for at least 60 consecutive days. Data collected include comprehensive clinical, functional and resource utilization information that are used to inform client needs. When used over time, it provides the basis for an outcome-based assessment of the person's response to care or services.
Registered Persons Database(RPDB)	The RPDB provides basic demographic information (age, sex, location of residence, date of birth, and date of death for deceased individuals) for those issued an Ontario health insurance number. The RPDB also indicates the time periods for which an individual was eligible to receive publicly funded health insurance benefits and the best known postal code for each registrant on July 1st of each year.
Discharge Abstract Database(DAD)	The DAD is compiled by the Canadian Institute for Health Information and contains administrative, clinical (diagnoses and procedures/interventions), demographic, and administrative information for all admissions to acute care hospitals, rehab, chronic, and day surgery institutions in Ontario. At ICES, consecutive DAD records are linked together to form 'episodes of care' among the hospitals to which patients have been transferred after their initial admission.
