# Supplementary material for: Associations between continuity of primary and specialty physician care and use of hospital-based care among community-dwelling older adults with complex care needs
Source: PLoS One. 2020 Jun 19;15(6):e0234205. doi: 10.1371/journal.pone.0234205 (PMC7304563; doi:10.1371/journal.pone.0234205)
Supplement: S2 Appendix — (PDF) [file pone.0234205.s002.pdf]

Appendix 2: Formulae, empirical example, and proof regarding the Bice-Boxerman and modified Bice-Boxerman continuity of care indices

Let n_i be the number of visits to ith provider and n_j be the number of visits within the jth specialty. The overall number of visits, number of providers, number of specialties are given by n, p, and s respectively.

The Bice-Boxerman continuity of care index is given by:

$$\frac{\left(\sum_{i=1}^{p} n_i^2\right) - n}{n^2 - n}$$

The modified Bice-Boxerman continuity of care index used in this study is defined as:

$$\frac{\left(\sum_{i=1}^{p} n_i^2\right) - n}{\left(\sum_{j=1}^{s} n_j^2\right) - n}$$

The modified Bice-Boxerman continuity of care index assumes that providers belong to one and only one specialty.

Empirical Example

Figure 1: Behavior of the original and modified Bice-Boxerman indices with increasing visits to multiple specialties

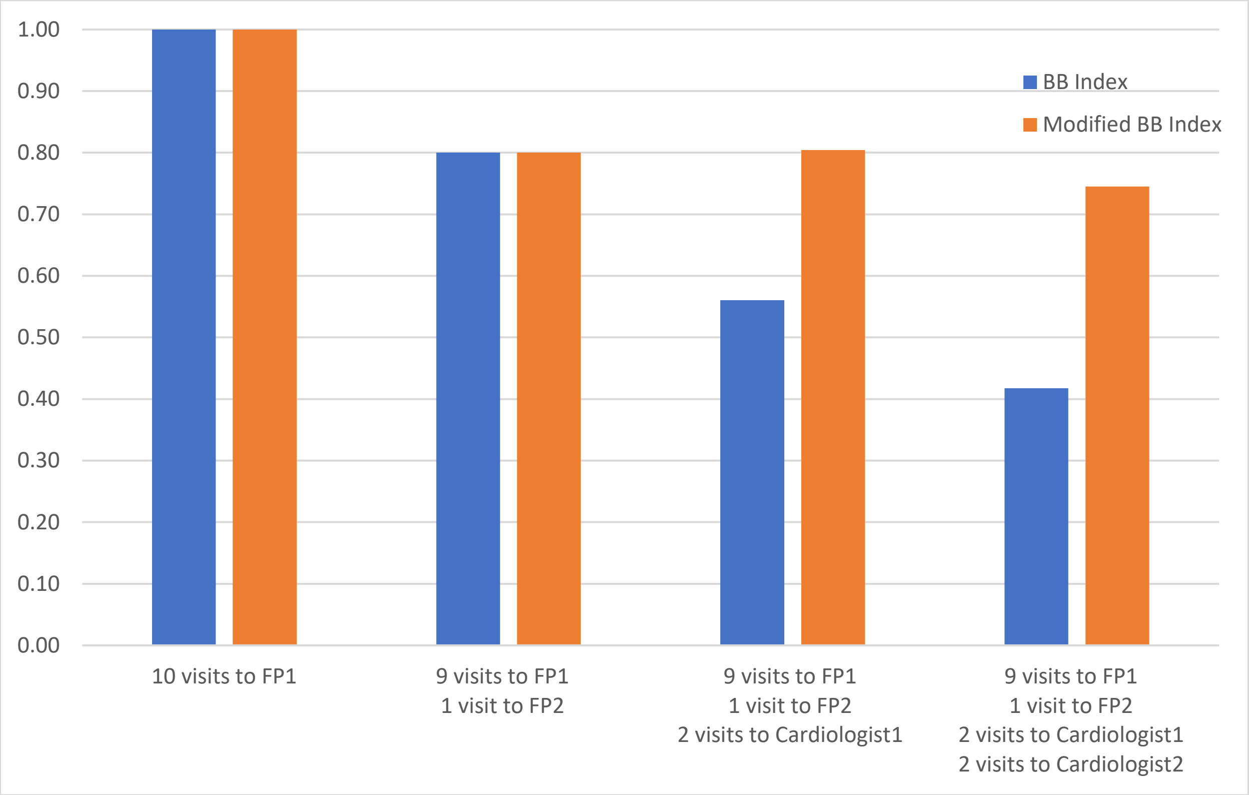

The above figure displays the value of the Bice-Boxmeran and modified Bice-Boxerman continuity indices under several scenarios involving visits to family physicians (FP) and cardiologists. The first set of bars representing a patient visiting the same family physician 10 times while the second set of bars represents a patient visiting one family physician 9 times and a different family physician once. The third set of bars represents a patient visiting one family physician 9 times, a different family physician once, and the same cardiologist twice, and the last set of bars represents the patient visiting one family physician 9 times, a different family physician once, and two different cardiologists twice each. The original Bice-Boxerman index drops in value across every scenario, including when then patient sees

only a single cardiologist, while the modified Bice-Boxerman index only drops in value when visits within a specialty are dispersed among multiple providers.

Proof

Let $n_i$be the number of visits to ith provider, $n_j$be the number of visits within the jth specialty, and n_{jk} be the number of visits to kth provider within the jth specialty. The overall number of visits, number of providers, number of specialties, and number of providers within each specialty j are given by n, p, s, and r_j respectively.

Proof that the modified Bice-Boxerman (MBB) index is a weighted averaged of specialty-specific unmodified Bice-Boxerman indices (BB) where each specialty has the weight $(n_j^2 - n_j)/(\sum_{j=1}^{s}(n_j^2) - n_j)$:

Assuming that each provider exists within only one specialty and that each $n_j \geq 2$ then:

$$\boldsymbol{BB = \frac{\left(\sum_{i=1}^{p} n_i^2\right) - n}{n^2 - n}}$$

$$\boldsymbol{BB_j = \frac{\left(\sum_{k=1}^{r} n_{jk}^2\right) - n_j}{n_j^2 - n_j}}$$

$$\boldsymbol{MBB = \frac{\left(\sum_{i=1}^{p} n_i^2\right) - n}{\left(\sum_{j=1}^{s} n_j^2\right) - n}}$$

$$\boldsymbol{= \frac{\left(\sum_{j=1}^{s}\left(\sum_{k=1}^{r} n_{jk}^2\right)\right) - \sum_{j=1}^{s} n_j}{\left(\sum_{j=1}^{s} n_j^2\right) - n}}$$

$$= \frac{\left(\sum_{j=1}^{s}\left(\sum_{k=1}^{r} n_{jk}^{2}\right) - n_{j}\right)}{\left(\sum_{j=1}^{s} n_{j}^{2}\right) - n}$$

$$= \frac{\sum_{j=1}^{s}\left(\left(\left(\sum_{k=1}^{r} n_{jk}^{2}\right) - n_{j}\right)\left(\frac{n_{j}^{2} - n_{j}}{n_{j}^{2} - n_{j}}\right)\right)}{\left(\sum_{j=1}^{s} n_{j}^{2}\right) - n}$$

$$= \frac{\sum_{j=1}^{s}\left(\frac{\left(\left(\sum_{k=1}^{r} n_{jk}^{2}\right) - n_{j}\right)\left(n_{j}^{2} - n_{j}\right)}{n_{j}^{2} - n_{j}}\right)}{\left(\sum_{j=1}^{s} n_{j}^{2}\right) - n}$$

$$= \frac{\sum_{j=1}^{s}\left(BB_{j}\left(n_{j}^{2} - n_{j}\right)\right)}{\left(\sum_{j=1}^{s} n_{j}^{2}\right) - n}$$

$$= \frac{BB_{1}\left(n_{1}^{2} - n_{1}\right)}{\left(\sum_{j=1}^{s} n_{j}^{2}\right) - n} + \frac{BB_{2}\left(n_{2}^{2} - n_{2}\right)}{\left(\sum_{j=1}^{s} n_{j}^{2}\right) - n} + \cdots + \frac{BB_{s}\left(n_{s}^{2} - n_{s}\right)}{\left(\sum_{j=1}^{s} n_{j}^{2}\right) - n}$$
