# Supplementary material for: Associations between continuity of primary and specialty physician care and use of hospital-based care among community-dwelling older adults with complex care needs
Source: PLoS One. 2020 Jun 19;15(6):e0234205. doi: 10.1371/journal.pone.0234205 (PMC7304563; doi:10.1371/journal.pone.0234205)
Supplement: S1 Document — (DOCX) [file pone.0234205.s003.docx]

| Project InitiationThis Section must be Completed Prior to Project Dataset(s) Creation | | | | | |
| --- | --- | --- | --- | --- | --- |
| **Project Title:** | Association of primary care characteristics and ED utilization among home care patients | | | | |
| **Project TRIM number:** | P0908.020.001 | | | | |
| **Research Program:** | HSPE | | | | |
| **Site:** | ICES McMaster | | | | |
| **Project Objectives:** | *Insert Project Objectives as listed in the approved ICES Project PIA* | | | | |
|  | To examine the associations between primary care characteristics and ED utilization among home care patients | | | | |
| **ICES Project PIA Initial Approval Date:** | *The ICES Employee or agent who is responsible for creating the Project Dataset(s) is responsible for ensuring there is an approved ICES Project PIA and verifying the date of approval prior to creating the Project Dataset(s)* | | | | |
|  | 2018-05-28 | | | | |
| **Principal Investigator (PI):** | Aaron Jones | | | | |
| **Check the applicable box if the PI is an ICES Student/Trainee** | ICES Student  ICES Fellow  ICES Post-Doctoral Trainee  Visiting Scholar | | | | |
| **Responsible ICES Scientist:** | *Name the Responsible ICES Scientist if the PI is not a Full Status ICES Scientist* | | | | |
|  | Dr. Susan Bronskill | | | | |
| **Project Team Member(s) Responsible for Project Dataset Creation and/or Statistical Analysis and date joined (list all):** | *All person(s) (ICES Analyst, Appointed Analyst, Analytic Epidemiologist, PI, and/or Student) responsible for creating the Project Dataset(s) and/or statistical analysis on the Research Analytics Environment (RAE) and the date they joined the project must be recorded* | | | | |
|  | Aaron Jones | | | 2017-11-20 | |
| **Other ICES Project Team Members and date joined (list all):** | *All other Research Project Team Members (e.g., Research Administrative Assistants, Research Assistants, Project Managers, Epidemiologists) and the date they joined the project must be recorded* | | | | |
|  | Richard Perez  Urun Erbas Oz  Ahmad Rahim | | | 2018-05-28  2018-05-28  2018-08-07 | |
| **Confirmation that DCP is consistent with Project Objectives:** | *The following individuals must confirm that the ICES Data provided for in this DCP is relevant (e.g., with respect to cohort, timeframe, and variables) and required to achieve the Project Objectives stated in the ICES Project PIA prior to initial Project Dataset creation: 1) PI; 2) Responsible ICES Scientist if the PI is not a Full Status ICES Scientist, or a second ICES Scientist or the Scientific Program Lead if the PI is creating both the DCP and the Project Dataset[s]; 3) ICES Research and Analysis Staff creating the DCP; and 4) ICES Analytic Staff (ICES Employee or agent responsible for creating the Project Dataset[s]). This may be delegated either verbally or via e-mail.* | | | | |
|  | ***Principal Investigator*** | |  | | 2018-12-07 |
|  | ***Responsible ICES Scientist or Second ICES Scientist/Lead*** | |  | 2018-12-07 | |
|  | ***ICES Research and Analysis Staff Creating the DCP*** | |  | yyyy-mon-dd | |
|  | ***ICES Analytic Staff*** | |  | 2018-12-07 | |
| **Designated ICES Research and Analysis Staff accountable for Project Documentation:** | *The person named (ICES staff) is accountable for ensuring that the approved ICES Project PIA, ICES Project PIA Amendments, and DCP are saved on the T Drive, ensuring ICES Project PIA Amendments are submitted as required, ensuring DCP Amendments are documented, and sharing the final DCP with the PI/Responsible ICES Scientist at project completion* | | | | |
|  |  | | | | |
| **DCP Creation Date and Author:** | *Date DCP was finalized prior to Project Dataset(s) creation* | *Name of person who created the DCP* | | | |
|  | ***Date*** | ***Name*** | | | |
|  | 2018-12-07 | Aaron Jones | | | |

| ICES DataThis Section must be Completed Prior to Project Dataset(s) Creation | |
| --- | --- |
| *The ICES Employee or agent who is responsible for creating the Project Dataset(s) must ensure that this list includes only data listed in the ICES Project PIA*  *Changes to this list after initial ICES Project PIA approval require an ICES Project PIA Amendment* | *Mandatory for all datasets that are available by individual year* |
| ***General Use Datasets – Health Services*** | ***Years (where applicable)*** |
| HCD | 2014-2017 |
| RAIHC | 2014-2016 |
| OHIP | 2012-2017 |
| NRS | 2013-2017 |
| CCRS | 2013-2017 |
| CIHI DAD | 2013-2017 |
| NACRS | 2013-2017 |
| OMHRS | 2013-2017 |
| ***General Use Datasets – Care Providers*** |  |
| CPDB | 2013-2017 |
| IPDB | 2013-2017 |
| ***General Use Datasets – Population*** |  |
| See list |  |
| See list |  |
| ***General Use Datasets – Coding/Geography*** |  |
| See list |  |
| See list |  |
| ***General Use Datasets - Facilities*** |  |
| See list |  |
| ***General Use Datasets - Other*** |  |
| CAPE | 2014-2017 |
| See list |  |
| ***Controlled Use Datasets*** |  |
| See list |  |
| See list |  |
| ***Other Datasets*** |  |
|  |  |

| Project Amendments and Reconciliation | | | |
| --- | --- | --- | --- |
| **ICES Project PIA Amendment History (add additional rows as needed):** | *Privacy approval date* | *Person who submitted amendment* | *Note that any changes to the list of ICES Data or Project Objectives require an ICES Project PIA Amendment* |
|  | ***Date*** | ***Name*** | ***Amendment*** |
|  | yyyy-mon-dd |  |  |
| **DCP Amendment History (add additional rows as needed):** | *Date DCP amended* | *Person who made the DCP amendment* | *Note that any DCP amendments involving changes to the list of ICES Data or Project Objectives require an ICES Project PIA Amendment* |
|  | ***Date*** | ***Name*** | ***Amendment*** |
|  | yyyy-mon-dd |  |  |
| **Date Programs/DCP reconciled** | *The person(s) creating the dataset and/or analyzing the data are responsible for ensuring that the final DCP reflects the final program(s) when the project is completed* | | |
|  | yyyy-mon-dd | | |

| Project Cohort | | |
| --- | --- | --- |
| **Study Design** | Cohort study  Matched cohort study  Case-control study  Cross-sectional study  Other (specify): | |
| **Index Event / Inclusion Criteria** | - Community RAI HC assessment belonging to a long-stay, home care patients with an assessment reference date between October 1st, 2014 and September 30th, 2016   - Assessment reference date (A1) is the index event date.   - If more than one assessment exists in the time period for an individual, choose the **last** assessment for cohort entry.   ***Inclusion Criteria:**   - RAI HC:   - Assessment reference date (A1) between October 1st, 2014 and September 30th, 2016   - Community assessments (RAI HC section H1aa – H1GB not blank)     - This should exclude ~ 10% of assessments     - Please exclude these first and then pick last assessment in window per IKN - HCD Clients:   - Assessment date (A1) occurred within a home care referral (REFERRAL_DATE <= a1 <= DDATE) that was admitted (ADMDATE > .)   Note: It is possible that a patient may have two admitted referrals at the time of assessment due to referrals in multiple LHINs. If this occurs choose the referral wheren the LHIN on the assessment is equal to the LHIN of the referral.  If there are still duplicate after this please select the last ADMDATE | |
| **Estimated Size of Cohort**  **(if known)** | ~200,000 | |
| **Exclusions (in order)** | *Step* | Description |
|  | 1 | Invalid IKN |
|  | 2 | Age < 19 at time of index assessment |
|  | 3 | Non-ontario resident |
|  | 4 | Dead before index |
|  | 5 |  |
|  | 6 |  |

| Project Time Frame Definitions | | |
| --- | --- | --- |
| Look-back Window  Observation Window  (in which to look for outcomes)  **Index Event Date**  Accrual Window  Max Follow-up Date | |  |
| **Accrual Start/End Dates** | October 1^st^, 2014-September 30^th^, 2016 |  |
| **Max Follow-up Date** | March 31^st^, 2017 |  |
| **When does observation window terminate?** | 6 months (182 days) following RAI HC assessment (index)  A1 + 182 |  |
| **Lookback Window(s)** | **A1-720 (Max look-back = October 1^th^, 2012)**   - Emergency department visits (NACRS) - Acute care inpatient admissions (DAD) - Physician visits (OHIP) - NRS - OMHRS - CCRS_LTC - CCRS |  |

| Variable Definitions (add additional rows as needed) | | |
| --- | --- | --- |
| **Main Exposure or Risk Factor** | **Dataset 1: Base cohort**  Baseline information on all cohort members at time of entry.   - IKN - Admit_date: (HCD ADMDATE) - Ref_Date: (HCD REFFERAL_DATE) - Disch_date: (HCD DDATE) - Disch_disp: (HCD DISCHDISP) - Ref_ID (HCD REFERRAL_IDENTIFIER) - Ref_source (HCD REFERRAL_SOURCE) - SRC_Admission (HCD: SRC_ADMISSION) - Sex: (RPDB) - Age: ( RPDB (Age in years as of index date)) - Living_arrangement: (HCD LIVING_ARRANGEMENTS) - Res_type: (HCD RESIDENCE_TYPE) - LHIN: (HCD LHIN) - FSA: RPDB - RIO: RPDB - Assessment_id_HC (linkable to index RAI-HCs) - PC_program: (CAPE: PROGTYPE) - FHT: (CPDB: Flag if grpnum is part of an FHT on index date) - Physnum_cape: (CAPE: Physnum of rostered PC provider) - Grpnum: (CAPE: GRPNUM of rostered provider) - Physnum_ohip: (provider with most visits) - Deathdate (RPDB: Date of death) - a1 as indexdate - marital status (married vs others. Created by using the variable BB4 from RAIHC) - MENTAL_HEALTH_WORKER_OP (FHT_Comp) - NURSE_PRACTITIONER_OP (FHT_Comp) - PHARMACIST_OP(FHT_Comp)   **Dataset 2: Index RAI-HC Assessments**  Assessment data from index assessments   - All fields from RAI-HC - a1 as indexdate   **Dataset 3: HCD Services**  Contains all service records of cohort members between six months before the index date and end of followup (>=A1 – 365 and <= A1 + 182)   - CARE_AUTH_ID - CARE_MODE - CARE_SITE - IKN - LHIN - REFERRAL_IDENTIFIER - SERVDATE - SERVICE - SERVICE_IDENTIFIER - SERVICE_RPC - UNIT_TIME - Weekend_holiday(flag for weekend or holiday based on servdate) - a1 as indexdate - max_follow_up   **Dataset 4: HCD Authorization**  Contains all authorizations that overlap with follow-up period (i.e. care_auth_date <= (A1+182) and (care_disch_date >= A1 or care_disch_date is null))   - CARE_AUTH_DATE - CARE_AUTH_ID - CARE_DISCH_DATE - CARE_SERVICE_TYPE - LHIN - REFERRAL_IDENTIFIER - a1 as indexdate - max_follow_up   **Dataset 5: OHIP**  Contains all OHIP records, ***excluding labs***, of cohort members between 720 days ***before*** the index date and the end of follow-up (A1 + 182). Link to IPDB to get MAINSPECIALTY   - DXCODE - EXPLAIN_CODE - FEECODE - FEESUFF - GRPNUM - IKN - LOCATION - NUMSERV - PAYTYPE - PHYSNUM - REFPHYS - SERVDATE - SPEC - MAINSPECIALTY (IPDB) - Weekend_holiday(flag for weekend or holiday based on SERVDATE) - a1 as indexdate - max_follow_up_ - lookback   **Dataset 6: ED Visits**  Contains all ED visits between 365 days before the index date and the end of follow-up (A1 + 182)   - IKN - ADMAMBUL - COMPLAINT1-3 - LEFTEDDATE - LEFTEDTIME - DX10CODE1 - DXCLUSTER1 - EDDISCHDX1 - INCODE1 - LOS_HRS - REGDATE - REGTIME - TRIAGE - TRIAGEDATE - TRIAGETIME - VISDISP2005 - Weekend_holiday(based on regdate) - a1 as indexdate - max_follow_up - lookback   **Dataset 7: DAD admissions**  Contains all DAD admits between 365 days before the index date and the end of follow-up (A1 + 182)   - IKN - ALCLOS - ADMAMBUL - ADMCAT - ADMDATE - ADMTIME - DDATE - DISCHDISP - DTIME - DX10CODE1 - DXCLUSTER1 - INCODE1 - INST - INSTTYPE - Weekend_holiday(flag for weekend or holiday based on admdate) - a1 as indexdate - max_follow_up_ - lookback   **Dataset 8: CCRS_LTC**  Contains all ccrs_ltc admits between 365 days before the index date and the end of follow-up (A1 + 182)   - IKN - ADMDATE - DDATE - AD2_Admission - Weekend_holiday(flag for weekend or holiday based on admdate) - a1 as indexdate - max_follow_up_ - lookback   **Dataset 9: CCRS**  Contains all ccrs admits between 365 days before the index date and the end of follow-up (A1 + 182)   - IKN - ADMDATE - DDATE - AD2_Admission - Weekend_holiday(flag for weekend or holiday based on admdate) - a1 as indexdate - max_follow_up_ - lookback   **Dataset 10: NRS**  Contains all nrs admits between 365 days before the index date and the end of follow-up (A1 + 182)   - IKN - ADMDATE - DDATE - Weekend_holiday(flag for weekend or holiday based on admdate) - a1 as indexdate - max_follow_up_ - lookback   **Dataset 11: OMHRS**  Contains all omhrs admits between 365 days before the index date and the end of follow-up (A1 + 182)   - IKN - ADMDATE - DDATE - Weekend_holiday(flag for weekend or holiday based on admdate) - a1 as indexdate - max_follow_up_ - lookback |  |
| **Primary Outcome Definition** | Emergency department admission within 6 months  Hospital admission within 6 months |  |
| **Secondary Outcome Definition(s)** |  |  |
| **Baseline Characteristics** | RAI-HC clinical data items, home care characteristics, retrospective health service use. |  |
| **Other Variables** |  |  |

| Analysis Plan and Dummy Tables (expand/modify as needed) | | |
| --- | --- | --- |
| **Descriptive Tables (insert or append dummy tables), e.g.:** | | |
| **Table 1: Baseline Characteristics** | | |
| **Table 2: Distribution of continuity of care** | | |
| **Table 3: Hazard ratios from Cox Proportional Hazard Models** | | |
| **Statistical Model(s)** | | |
| **Type of model** | Cox proportional hazards models | |
| **Primary independent variable** | Continuity of care | |
| **Dependent variable** | ED Visits / Hospital admissions | |
| **Covariates** | RAI-HC clinical data items, home care characteristics | |
|  | |  |

| Quality Assurance Activities | | | |
| --- | --- | --- | --- |
| **RAE Directory of SAS Programs** |  | | |
| **RAE Directory of Final Dataset(s)** | *The* *final analytic dataset for each cohort includes all the data required to create the baseline tables and run all the models. It should include all covariates for all models such as patient risk factors, hospital characteristics, physician characteristics, exposure measures (continuous, categorical) and outcomes. It should include covariates that were considered but didn’t make the final cut. This would permit an analyst to easily re-run the models in the future.* | | |
|  |  | | |
| **RAE README file available:** Yes No | | | |
| **Date results of quality assurance tools for final dataset shared with project team (where applicable):** | | |  |
|  | | **%assign** | yyyy-mon-dd |
|  | | **%evolution** | yyyy-mon-dd |
|  | | **%dinexplore** | yyyy-mon-dd |
|  | | **%track / %exclude** | yyyy-mon-dd |
|  | | **%codebook** | yyyy-mon-dd |
| **Additional comments:** | |  | |
